# Supplementary material for: Locating causal hubs of memory consolidation in spontaneous brain network in male mice
Source: Nat Commun. 2023 Sep 5;14:5399. doi: 10.1038/s41467-023-41024-z (PMC10480429; doi:10.1038/s41467-023-41024-z)
Supplement: Supplementary file 1 — Supplementary Information [file 41467_2023_41024_MOESM1_ESM.pdf]

Supplementary Information

Supplementary Results

Behavioral results of 1-Day APA and 5-Day APA

Over five 10 min training trials, the number of shocks (N_{shock}) that animals received, which represents a failure to avoid the shock zone, gradually decreased in both tasks ($F_{4, 36} = 10.27$, $p < 0.0001$ for 1-Day APA, $F_{4, 28} = 7.11$, $p = 0.0004$ for 5-Day APA, one-way ANOVA; Fig. 1b). Compared to the last trial of training (T5), the N_{shock} was significantly lower than for the first three training trials, T1 ($t = 4.81$, $p < 0.0001$), T2 ($t = 4.81$, $p < 0.0001$) and T3 ($t = 3.97$, $p = 0.0002$), in the 1-Day APA. In the 5-Day APA, the N_{shock} of T5 was lower than for the first two training trials, T1 ($t = 4.56$, $p < 0.0001$) and T2 ($t = 3.62$, $p = 0.0005$). The time to first entrance into the shock zone (i.e., the initial avoidance time; T_{enter}) is another index of how well the memory is recalled in each trial. In the 1-Day APA, the T_{enter} was significantly longer in T5 compared to the first three training trials, T1 ($t = 2.97$, $p = 0.016$), T2 ($t = 2.93$, $p = 0.017$), and T3 ($t = 2.89$, $p = 0.018$). In the 5-Day APA, the T_{enter} in T5 was significantly longer than the first training trial ($t = 2.62$, $p = 0.034$). These results indicate successful learning in both tasks.

During the probe test, neither measure was significantly different from the last training trial in both the 1-Day (N_{shock}: $t = 1.36$, $p = 0.18$; T_{enter}: $t = 1.23$, $p = 0.25$) and 5-Day (N_{shock}: $t = 0.82$, $p = 0.42$; T_{enter}: $t = 1.76$, $p = 0.12$) APA, indicating good memory retention (Fig. 1a). Comparing the 1-Day and 5-Day APA, no difference was found in the N_{shock} ($F_{1, 16} = 0.55$, $p = 0.47$, two-way ANOVA) or T_{enter} ($F_{1, 16} = 1.27$, $p = 0.28$).

Group independent component analysis results

We performed group ICA with 30 components on the 1-Day APA datasets and used dual regression to test the difference between 1-Day APA vs 1-Day control on post-training days 1 and 8 individually. Fig. S1 shows the ICA components which had significantly changed brain regions. In general, large spatial components were distinguished that could be categorized as cortical, limbic, basal ganglia and thalamic networks.

On post-training day 1, post-encoding changes with four cortical network components were found (Fig. S1a). APA training significantly increased the connectivity between the thalamus and the somatosensory component, and the connectivity of the HPC, CPu and visual cortex with the visual component. In sensory processing networks, the connectivity between the HPC and the insular component, and between the Pons, PAG and the piriform component was increased. Among the six limbic networks, the connectivity of the Pons, LS and HPC with the RSC component, and

the connectivity of the LD, S1HL, M1 and ACC with the HPC component was decreased. On the contrary, the connectivity between the mPFC and the ACC component, the connectivity of the CPu and S1 with the ACC+RSC component, and the connectivity of the Hyp and IEn with the amygdala component were increased. In the two basal ganglia networks, the connectivity with the HPC, sensory, prefrontal, and basal ganglia was increased.

On post-training day 8, we detected similar but reorganized network components (Fig. S1b). In the sensory networks, decreased connectivity of the S1BF, mPFC, AcbC and dorsal thalamus with somatosensory components was found. In sensory processing networks, the connectivity of the MB and thalamus was increased whereas the connectivity of the CPu was decreased. In the limbic networks, the connectivity of the FrA, AcbC and VL with the RSC component was decreased. The connectivity of the HPC, thalamus and MD with the HPC component was decreased, as was the connectivity between the S1 with the amygdala component. In the basal ganglia networks, the connectivity with the thalamus was decreased while the connectivity with the sensory processing area (Ins) was increased. Finally, the connectivity within the thalamus was decreased.

Compared to the results of the seed-based analysis (Fig. 1d), ICA detected more changes on post-training day 1, including the RSC, amygdala, and basal ganglia networks. Consistent with the results of the seed-based analysis, both increased and decreased connectivity were found across the brain. ICA detected more increased FC on post-training day 1 and more decreased FC on post-training day 8. Similar regions could also be identified. However, the specific connections differed. This could be due to the very large and broad ICA component that typically includes multiple brain areas. It would therefore be difficult to detect small brain regions and to pinpoint a specific region involved in memory consolidation.

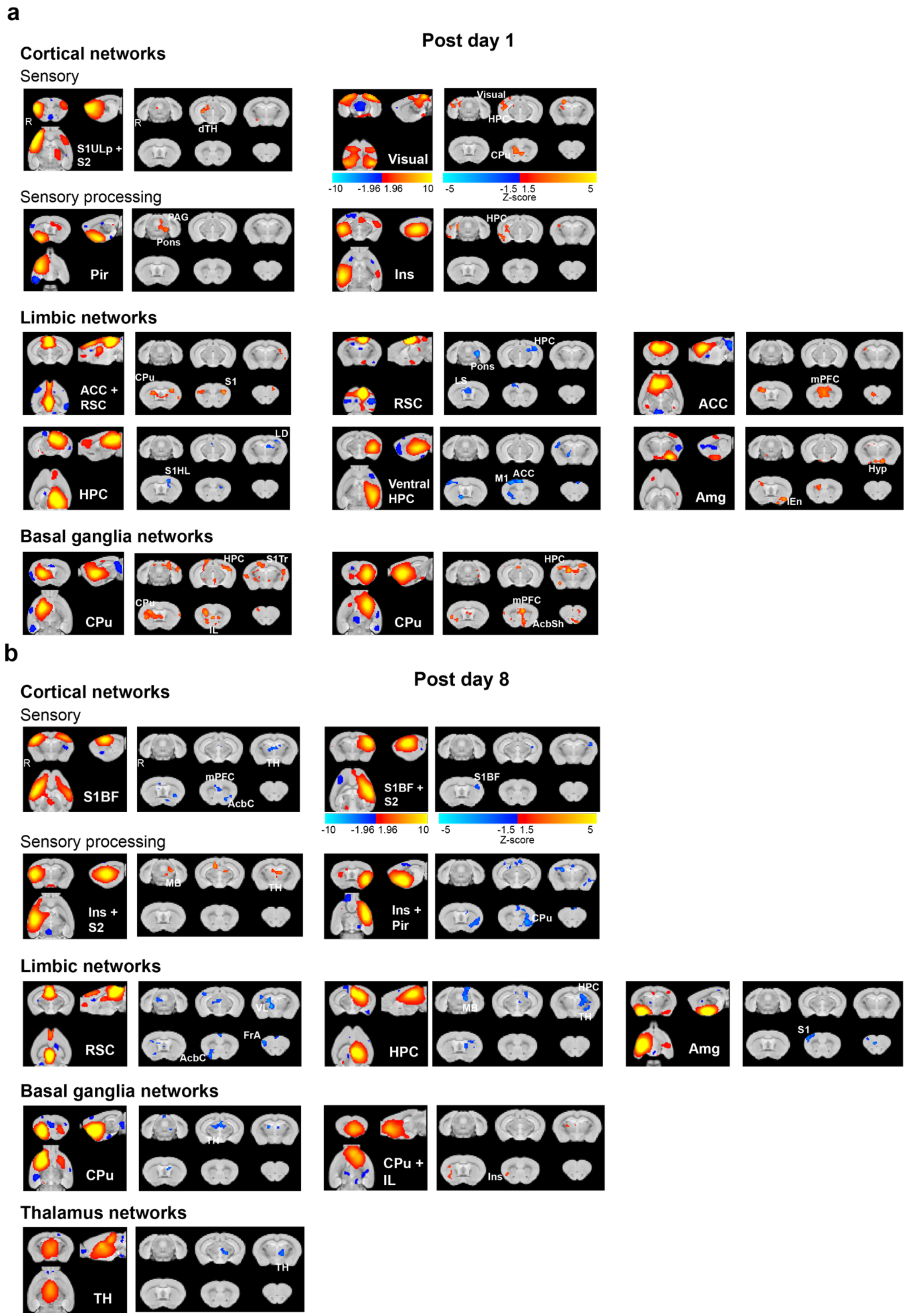

Fig. S1 Post-encoding RSN changes of 1-Day APA detected by group ICA.

ICA components which have significantly changed brain regions on (**a**) post-training day 1 and (**b**) post-training day 8, overlaid on the AMBMC atlas template (https://imaging.org.au/AMBMC/AMBMC). In each subgraph, the 3-plane view on the left shows the ICA component map, and the lightbox view on the right shows the connected brain regions detected by dual regression (APA vs control, $p<0.05$, two tail cluster-level corrected). AcbC, accumbens nucleus core; Acbsh, accumbens nucleus shell; ACC, anterior cingulate cortex; Amg, amygdala; BF, basal forebrain; CPu, caudate putamen; dTL, dorsal thalamus; FrA, frontal association cortex; HPC, hippocampus; Hyp, hypothalamus; IEn, intermediate nucleus of the endopiriform cortex; IL, infralimbic area; Ins, insular cortex; LD, laterodorsal thalamic nucleus ; LS, lateral septum; M1, primary motor cortex; MB, midbrain; mPFC, medial prefrontal cortex; PAG, periaqueductal gray; Pir, piriform cortex; RSC, retrosplenial cortex; S1, primary somatosensory cortex; S1BF, primary somatosensory cortex, barrel field; S1HL, primary somatosensory cortex, hindlimb region; S1Tr, primary somatosensory cortex, trunk region; S1ULp, primary somatosensory cortex, upper lip region; S2, secondary somatosensory cortex; TeA, temporal association area; TH, thalamus; VL, ventrolateral thalamic nucleus.

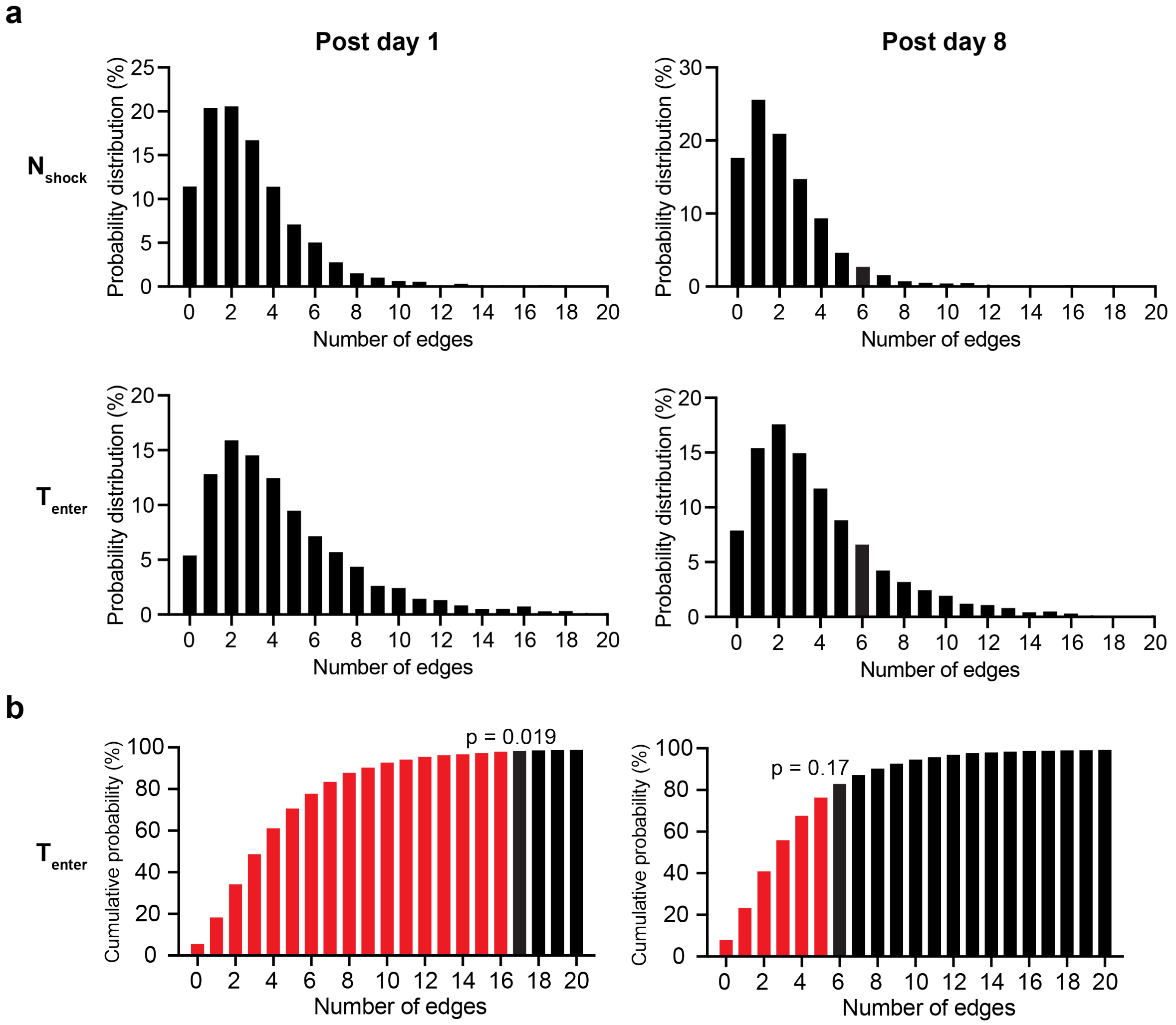

Fig. S2 Null distribution of the common networks.
(**a**) The null distribution of the number of edges when 5000 permutated networks of the 1-Day and 5-Day APA were thresholded at $p<0.05$ and correlated with N_{shock} (upper row) or T_{enter} (lower row) at $p<0.05$. (**b**) The cumulative probability of the null distribution in (**a**) when correlated with the T_{enter}. The red bars show the cumulative probability before reaching the real number of edges and the corresponding p values for the real networks based on the permutation tests. Source data are provided as a Source Data file.

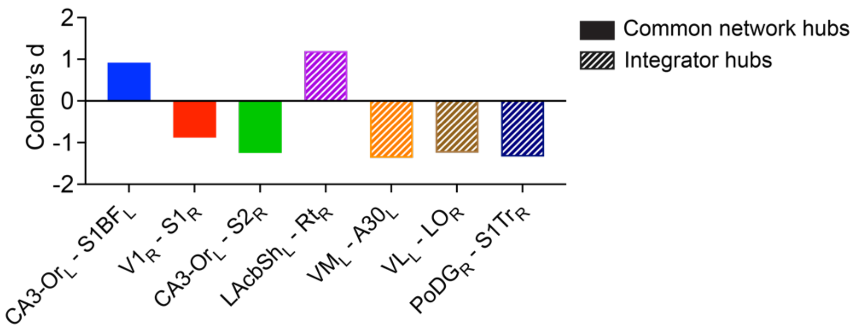

Fig. S3 The effect size of functional connections identified by the hub identification.
The first three functional connections were identified by common network analyses (Fig. 2d, e, Supplementary Table S2). The last five connections were identified by integrator hub analyses (Table 2). Source data are provided as a Source Data file.

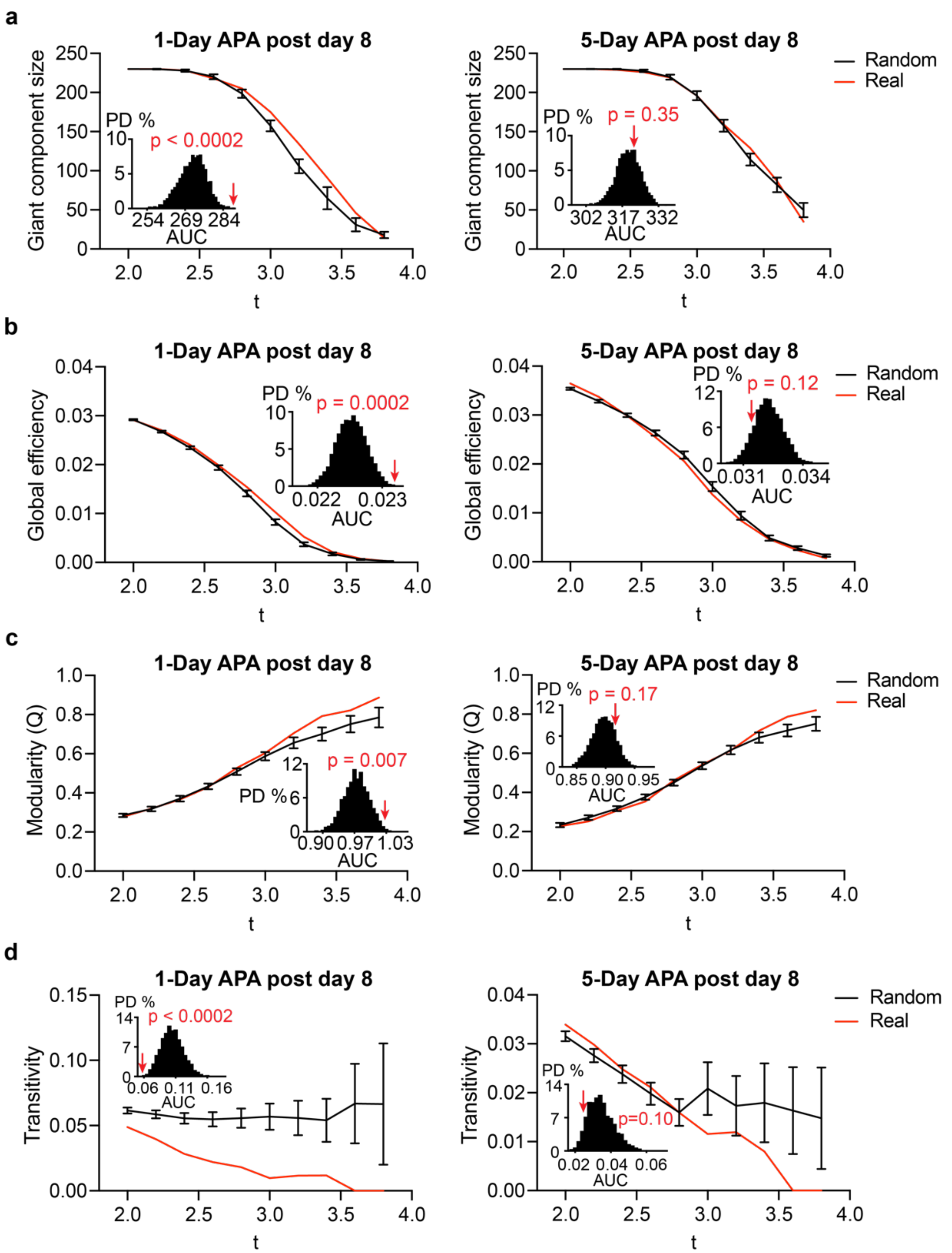

Fig. S4 Network graph properties of post-encoding RSNs on post-training day 8. Trends of (**a**) giant component size, (**b**) global efficiency, (**c**) modularity and (**d**) transitivity from the post-encoding RSNs (red) on post-training day 8 compared to random networks (black), thresholded at $2 \leq t \leq 3.8$. The black line shows the mean □ SD of 5000 random networks generated based on the real network. The embedded bar graphs show the null distribution of the area under curve (AUC) of the random networks, The red arrows indicate the value for the real network and the corresponding p value based on the permutation test. PD: probability distribution. Source data are provided as a Source Data file.

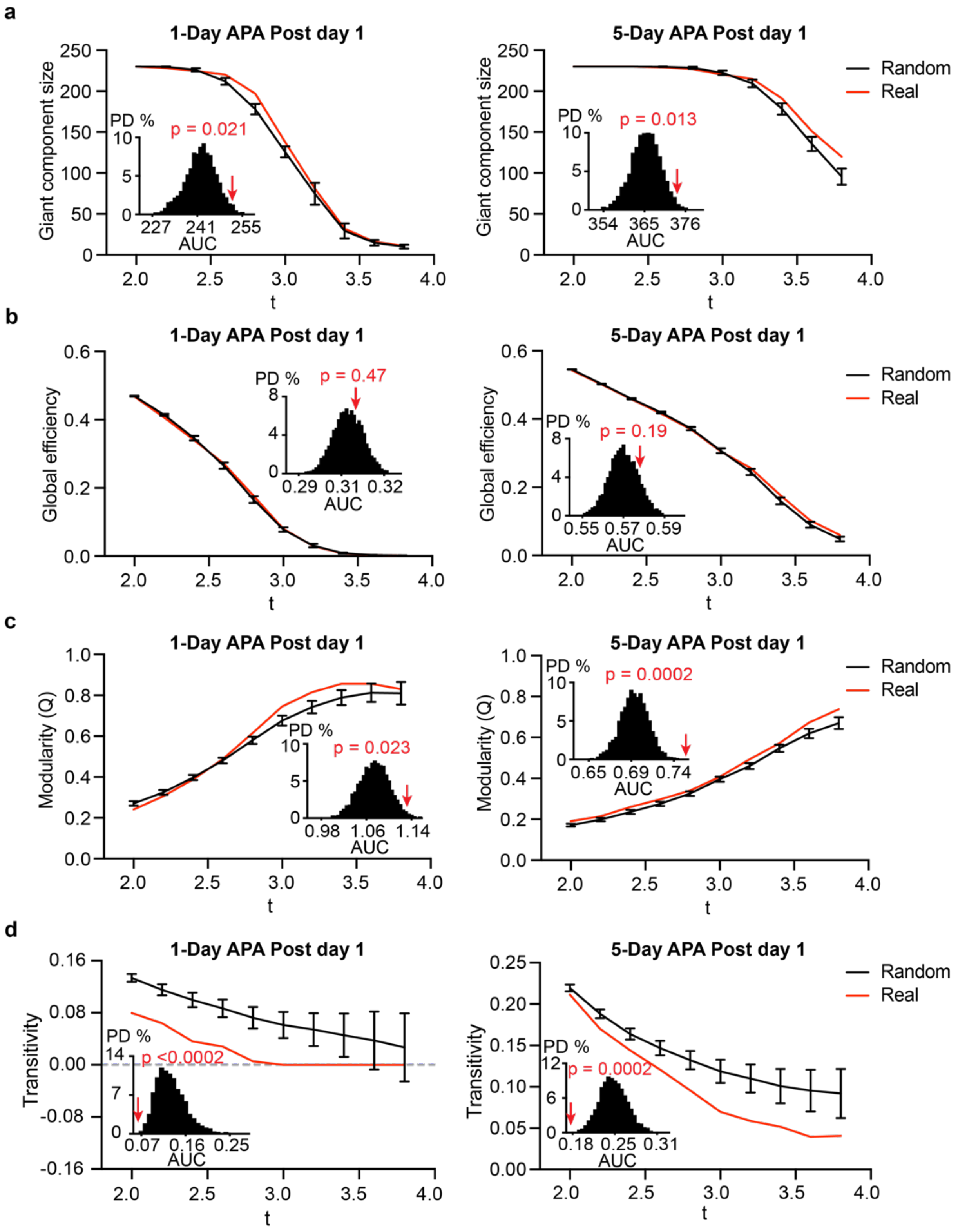

Fig. S5 Unweighted network graph properties of post-encoding RSNs on post-training day 1. Trends of (**a**) giant component size, (**b**) global efficiency, (**c**) modularity and (**d**) transitivity from the post-training day 1 (red) compared to random networks (black), thresholded at $2 \leq t \leq 3.8$. Unweighted FC matrices were used to generate the above plots. The black line shows the mean □ SD of 5000 random networks generated based on the real network. The embedded bar graphs show the null distribution of the area under curve (AUC) of the random networks, The red arrows indicate the value for the real network and the corresponding p value based on the permutation test. PD: probability distribution. Source data are provided as a Source Data file.

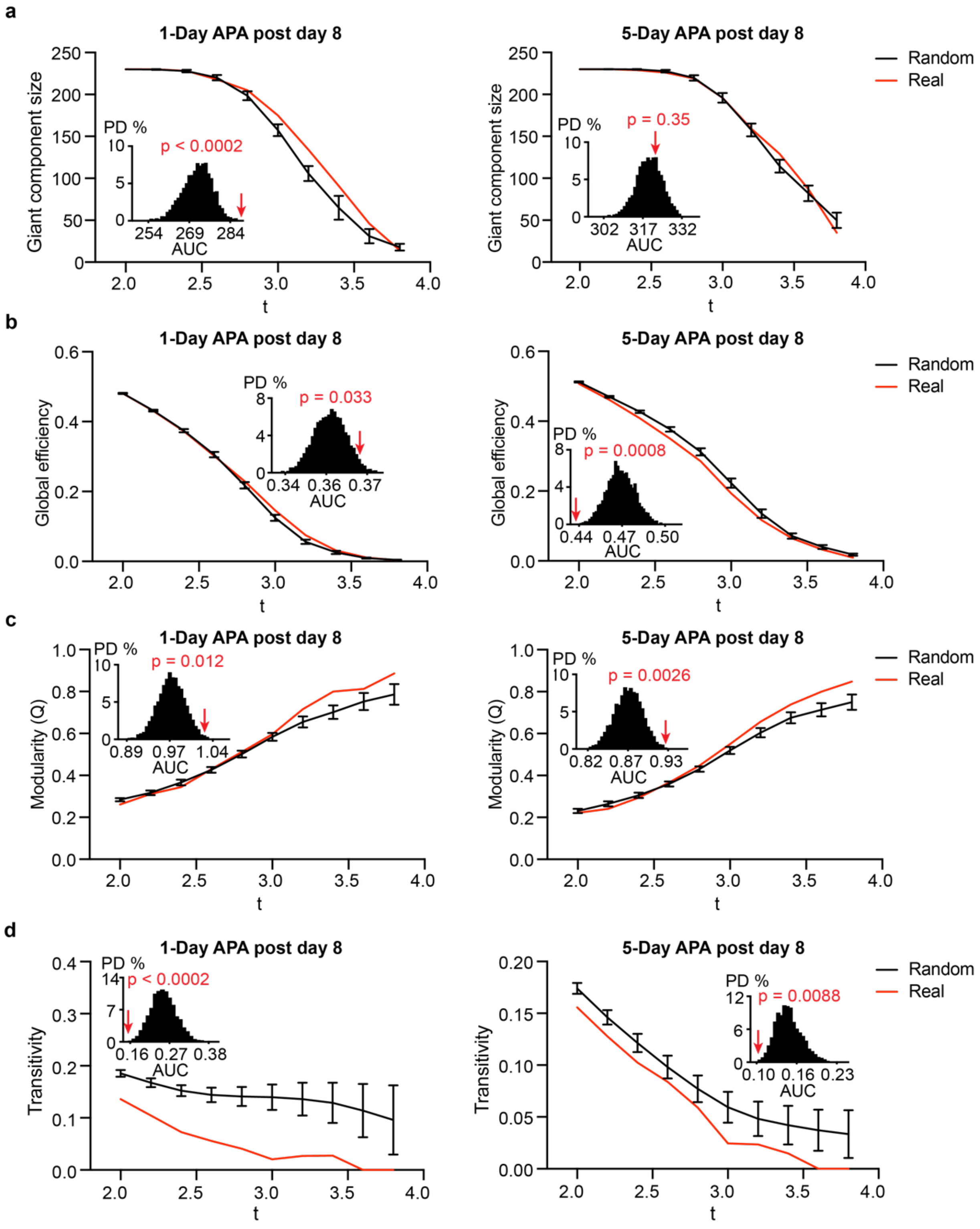

Fig. S6 Unweighted network graph properties of post-encoding RSNs on post-training day 8. Trends of (**a**) giant component size, (**b**) global efficiency, (**c**) modularity and (**d**) transitivity from the post day 8 (red) compared to random networks (black), thresholded at $2 \leq t \leq 3.8$. Unweighted FC matrices were used to generate the above plots. The black line shows the mean □ SD of 5000 random networks generated based on the real network. The embedded bar graphs show the null distribution of the area under curve (AUC) of the random networks, The red arrows indicate the value for the real network and the corresponding p value based on the permutation test. PD: probability distribution. Source data are provided as a Source Data file.

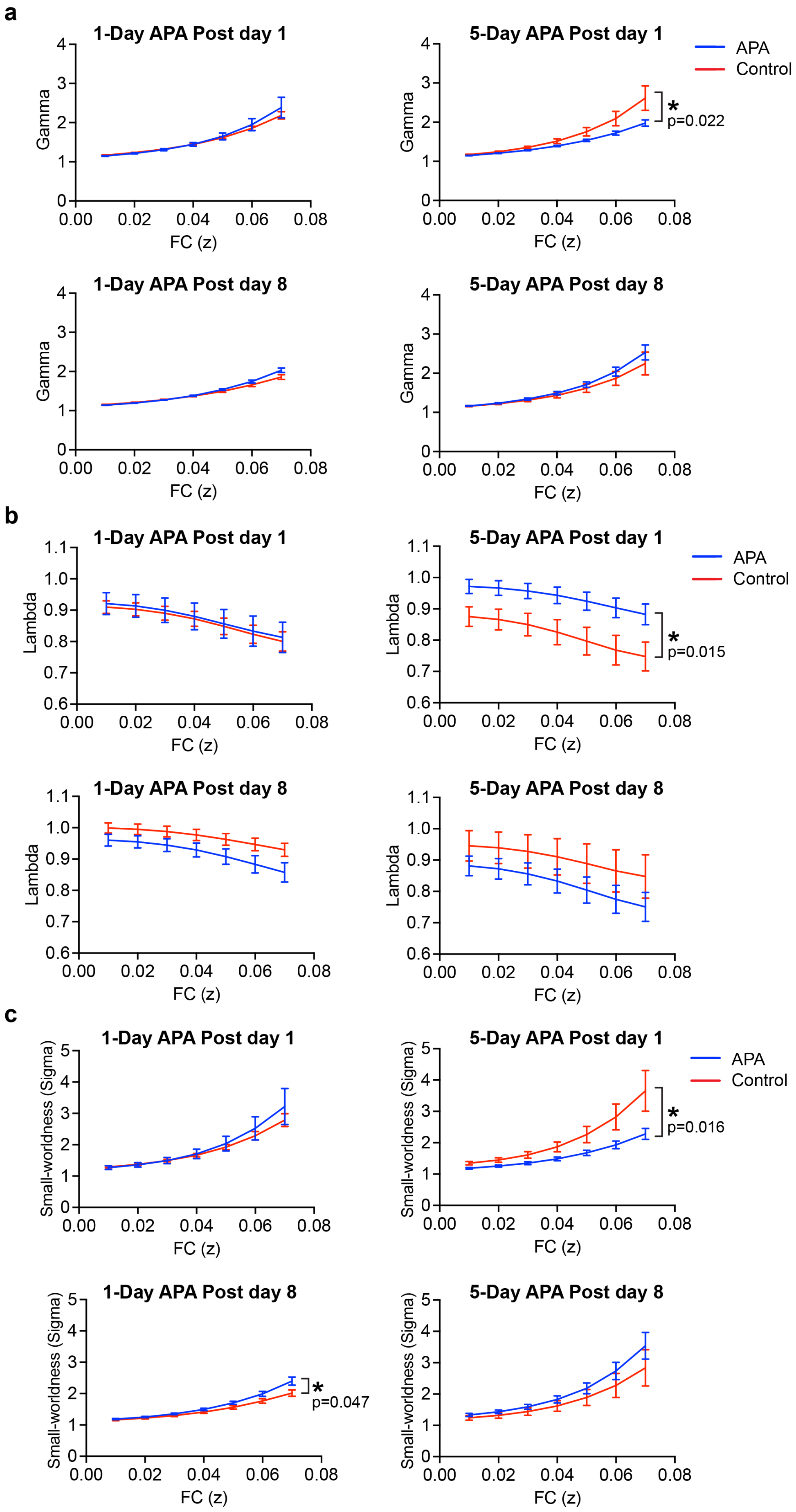

Fig. S7 Small-world network analysis of post-encoding RSNs.

Trends in (**a**) gamma, (**b**) lambda and (**c**) small-worldness (sigma) of the post-encoding RSNs thresholded at $0.01 \leq z \leq 0.07$. The maximum threshold selected was the value for which all FC matrices were fully connected (no isolated node). $z = 0.0254$ correspond to $p = 0.05$ for the Pearson's correlation between seed time-courses, and $z = 0.07$ correspond to $p<0.0000$, uncorrected. Data are presented as mean SEM. See Supplementary Table S4 for N of each group. The * indicates the significant difference between the AUC of the trends based on two-sample t-test, one-tailed. *: $p<0.05$. Source data are provided as a Source Data file.

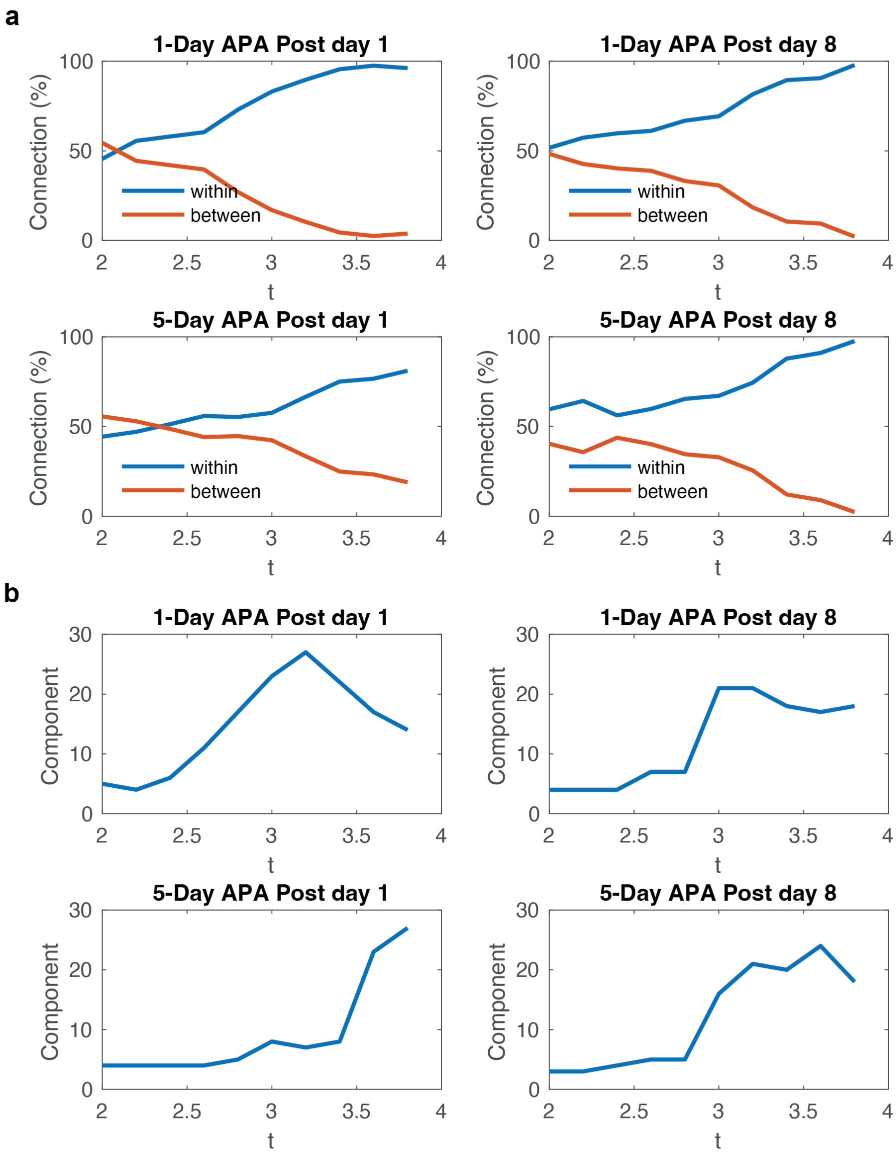

Fig. S8 Modular connectivity of post-encoding RSNs.
(**a**) The proportional connections within and between network components, and (**b**) the number of components (modules) over the network threshold at $2 \leq t \leq 3.8$.

a

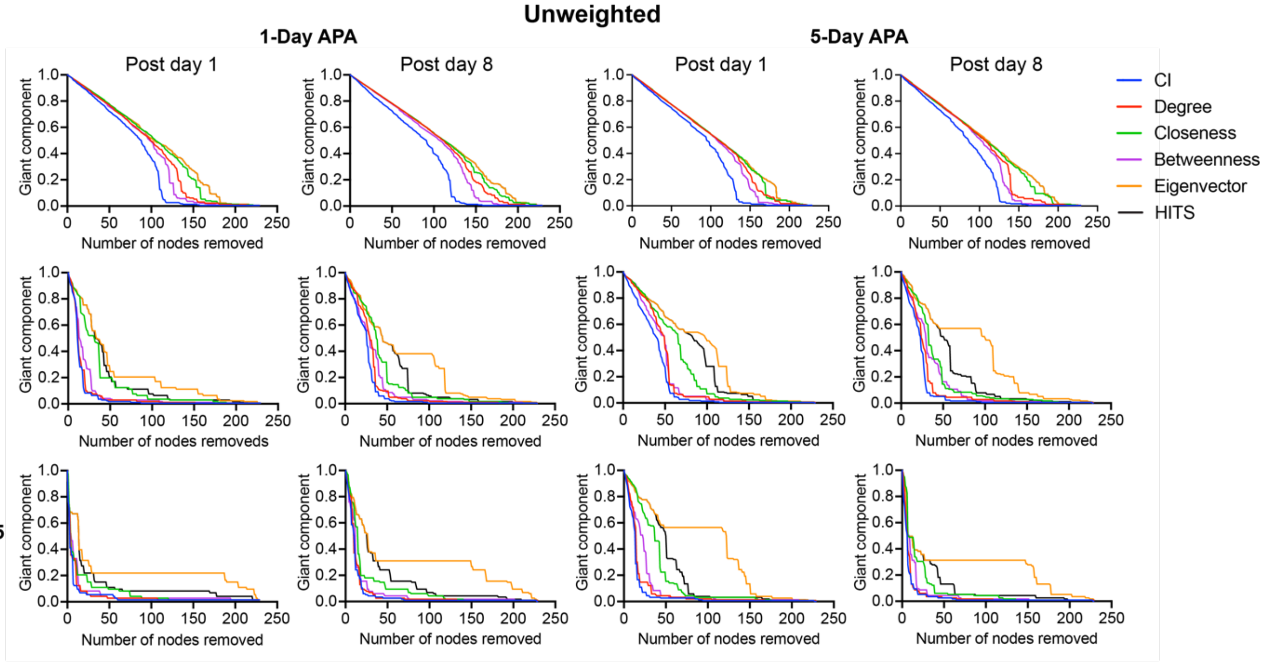

b

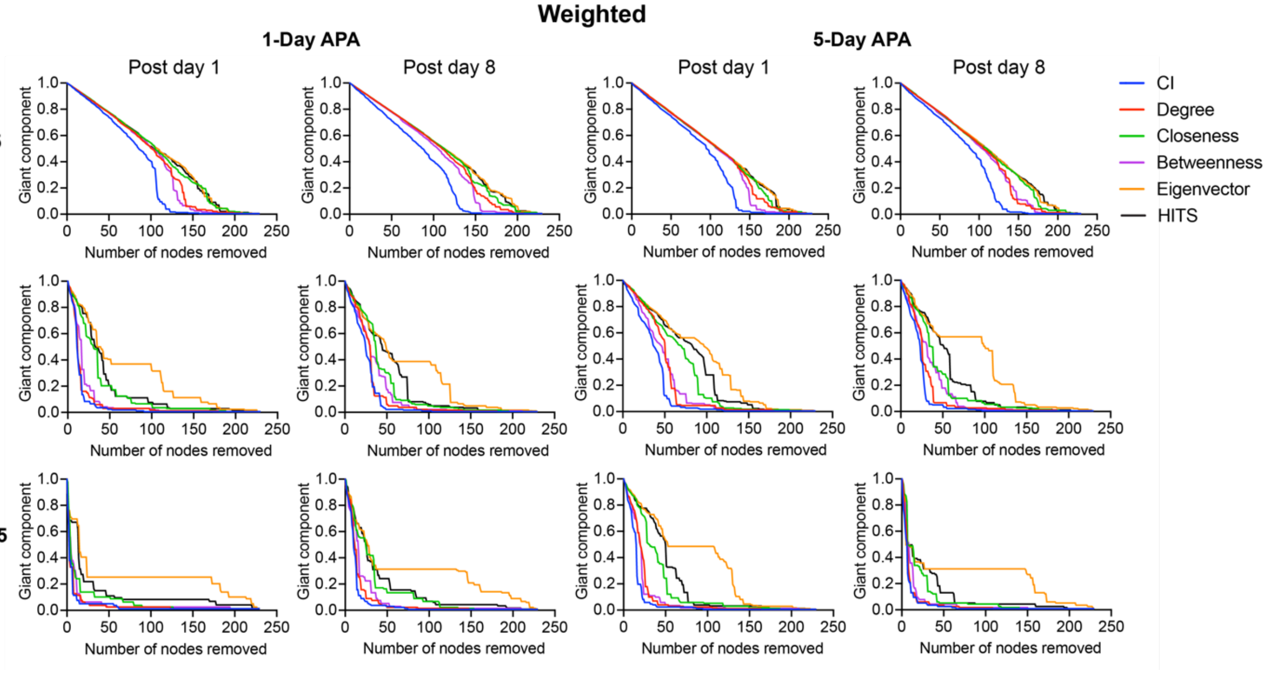

Fig. S9 Reduction of the giant component by removing hubs under different network thresholds.

Six hub selection methods were compared, including CI, degree centrality, closeness centrality, betweenness centrality, eigenvector centrality and HITS using either (**a**) unweighted or (**b**) weighted FC matrices (two-sample t-test, two-tailed, uncorrected). Note that CI and HITS can only use the unweighted FC matrix as input. Each line represents the proportional size of the remaining giant component after the nodes were removed from the network one by one according to the rank obtained using each method. Each row represents the results of the FC matrices threshold at $p<0.05$, 0.01 and 0.005, respectively. Source data are provided as a Source Data file.

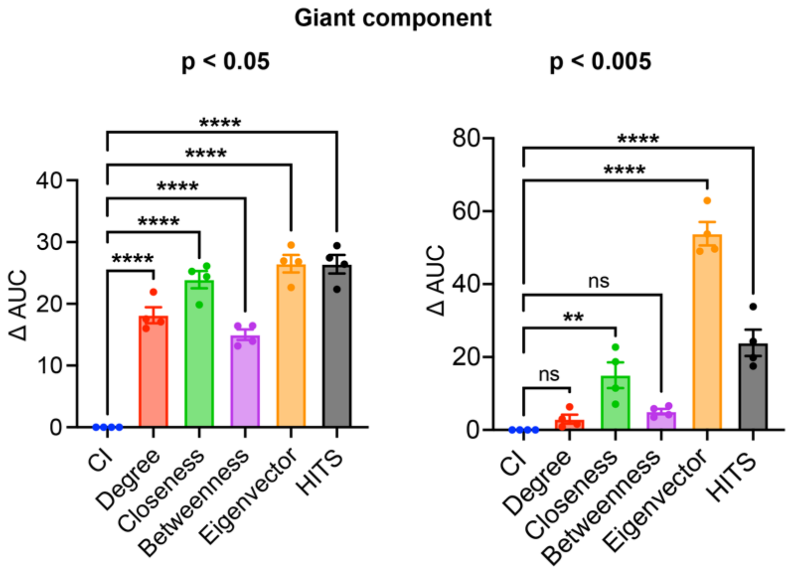

Fig. S10 Comparison of methods for reducing the giant component.
The change in area under the curve (ΔAUC) of the giant component reduction curves when the difference FC matrices were thresholded at $p<0.05$ (left) or 0.005 (right) (two-sample t-test, two-tailed, uncorrected). CI analysis shows the quickest reduction of giant components at both thresholds (left: $F_{5, 15} = 138.93$, $p = 5.18\times10^{-12}$; right: $F_{5, 15} = 73.56$, $p = 5.18\times10^{-10}$, one-way ANOVA). With a network threshold at $p<0.05$ (left), post hoc comparison (Bonferroni's multiple comparison test) with the CI method: degree, $p = 1.49\times10^{-9}$; closeness, $p = 2.88\times10^{-11}$; betweenness, $p = 1.25\times10^{-7}$; eigenvector, $p = 5.33\times10^{-12}$; HITS, $p = 5.33\times10^{-12}$. At $p<0.005$ (right): closeness, $p = 0.0090$; eigenvector, $p = 3.70\times10^{-10}$; HITS, $p = 2.73\times10^{-5}$. N = 4 experimental groups. Each data point was normalized by the corresponding AUC of CI. Data are represented as mean SEM. * $p < 0.05$; *** $p<0.001$; **** $p<0.0001$. Source data are provided as a Source Data file.

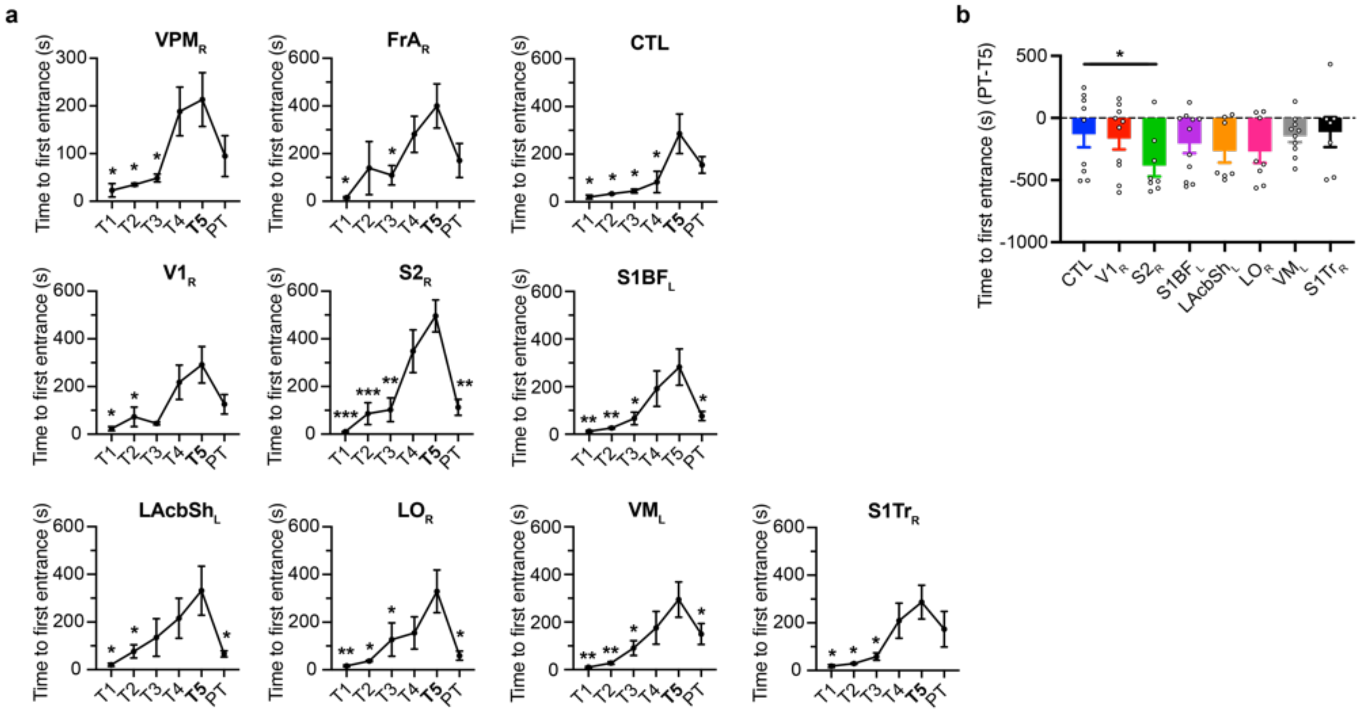

Fig. S11 The effect of hub inhibition on the T_{enter}.

(**a**) The T_{enter} during training (T1 to T5) and effect of DREADDs inhibition or CNO control (CTL) on the probe test (PT). Significant increasing trends were seen during learning for $V1_R$ (one-way ANOVA, $F_{5, 45} = 5.29$, $p = 0.0007$; T1, $p = 0.021$; T2, $p = 0.042$; N = 10), $S2_R$ ($F_{5, 35} = 12.36$, $p < 0.0001$; T1, $p = 0.00019$; T2, $p = 0.00074$; T3, $p = 0.0012$; PT, $p = 0.0032$; N = 8), $S1BF_L$ ($F_{5, 50} = 4.69$, $p = 0.0014$; T1, $p = 0.0052$; T2, $p = 0.0078$; T3, $p = 0.034$; PT, $p = 0.024$; N = 11), CNO-control (CTL; $F_{5, 40} = 5.92$, $p = 0.0003$; T1, $p = 0.013$; T2, $p = 0.016$; T3, $p = 0.027$; T4, $p = 0.025$; N = 9), $LAcbSh_L$ ($F_{5, 30} = 3.22$, $p = 0.019$; T1, $p = 0.026$; T2, $p = 0.032$; PT, $p = 0.030$; N = 7), LO_R ($F_{5, 35} = 4.53$, $p = 0.0027$; T1, $p = 0.0098$; T2, $p = 0.015$; T3, $p = 0.034$; PT, $p = 0.022$; N = 8), VM_L ($F_{5, 45} = 5.16$, $p = 0.0008$; T1, $p = 0.0044$; T2, $p = 0.0071$; T3, $p = 0.039$; PT, $p = 0.018$; N = 10), $S1Tr_R$ ($F_{5, 30} = 4.11$, $p = 0.0058$; T1, $p = 0.031$; T2, $p = 0.039$; T3, $p = 0.024$; N = 7), VPM_R ($F_{5, 20} = 6.13$, $p = 0.0013$; T1, $p = 0.017$; T2, $p = 0.035$; T3, $p = 0.046$; N = 5), and FrA_R ($F_{5, 20} = 4.45$, $p = 0.0069$; T1, $p = 0.013$; T3, $p = 0.022$; N = 5). All the post hoc comparisons were performed with respect to the last training trial (T5) by Dunnett's multiple comparison test. (**b**) The comparison of changed T_{enter} (PT – T5) between experimental groups and CTL shows a significant decrease when $S2_R$ is inhibited (two-sample t-test, one-tailed, $p = 0.044$, uncorrected). Data are represented as mean □ SEM. The number of animals represents biologically independent mice. * $p < 0.05$; ** $p < 0.01$; *** $p<0.001$. Source data are provided as a Source Data file.

Table S1 The ROIs used in seed-based FC analysis.
This ROIs list was used to create the FC matrix. The size of one voxel is 0.2 x 0.2 x 0.2 = 0.008 mm^3.

Major region	Abbreviation	Structure name	Number of voxels
**Medial prefrontal cortex (mPFC)**	A24a	Anterior cingulate area, ventral part	79
	A24a'	Anterior cingulate area, ventral part	40
	A24b	Anterior cingulate area, dorsal part	72
	A24b'	Anterior cingulate area, dorsal part	27
	IL	Infralimbic area	29
	PL	Prelimbic area	108
**Sensory-motor cortex**	Au1	Primary auditory cortex	65
	AuD	Secondary auditory cortex, dorsal area	65
	AuV	Secondary auditory cortex, ventral area	73
	M1	Primary motor cortex	340
	M2	Secondary motor cortex	284
	S1	Primary somatosensory cortex, unassigned	184
	S1BF	Primary somatosensory cortex, barrel field	433
	S1FL	Primary somatosensory cortex, forelimb region	179
	S1HL	Primary somatosensory cortex, hindlimb region	125
	S1J	Primary somatosensory cortex, jaw region	25
	S1Tr	Primary somatosensory cortex, trunk region	33
	S1ULp	Primary somatosensory cortex, upper lip region	207
	S2	Secondary somatosensory cortex	281
	V1	Primary visual cortex	112
	V1B	Primary visual cortex, binocular area	84
	V1M	Primary visual cortex, monocular area	89
	V2L	Secondary visual cortex: lateral area	107
	V2ML	Secondary visual cortex: mediolateral area	53
	V2MM	Secondary visual cortex: mediomedial area	74
**Other cortical areas**	A29a	Retrosplenial area, ventral part	34
	A29c	Retrosplenial area, ventral part	82
	A30	Retrosplenial area, dorsal part	106
	Amg	Amygdala	344
	APir	Amygdalopiriform transition area	47
	Ce	Central amygdaloid nucleus	41
	CxA	Cortex amygdala transition zones	33
	DEn	Dorsal nucleus of the endopiriform	53
	DTT	Dorsal tenia tecta	52
	Ect	Ectorhinal cortex	117
	FrA	Frontal association cortex	285

	IEn	Intermediate nucleus of the endopiriform claustrum	25
	Ins	Insular region, not subdivided	317
	LO	Lateral orbital cortex	185
	MeA	Medial amygdala	36
	MO	Medial orbital cortex	70
	MPtA	Medial parietal association cortex	20
	Pir	Piriform cortex	470
	PLCo	Posterolateral cortical amygdaloid area	35
	PMCo	Posteromedial cortical amygdaloid area	54
	PRh	Perirhinal cortex	95
	RAPir	Rostral amygdalopiriform area	17
	TeA	Temporal association area	114
	VCl	Claustrum, ventral part	23
	VO	Ventral orbital cortex	66
**Basal ganglia & associated areas**	AcbC	Accumbens nucleus core	83
	AcbSh	Accumbens nucleus shell	77
	CPu	Caudate putamen	962
	DB	Diagonal band	45
	EA	Extension of the amygdala	29
	GP	Globus pallidus	118
	IPAC	Interstitial nucleus of the posterior limb of the anterior commissure	33
	LAcbSh	Accumbens nucleus shell, lateral part	25
	LS	Lateral septum	136
	MS	Medial septal nucleus	30
	PBGN	Posterior basal ganglia nucleus	17
	ST	Bed nucleus of the stria terminalis	58
	Tu	Olfactory tubercle	132
	VP	Ventral pallidum	67
**Hippocampal formation (HPF)**	CA1-Lmol	CA1 lacunosum molecular layer	86
	CA1-Or	CA1 oriens layer	116
	CA1-Rad	CA1 radiatum layer	149
	CA2-Rad	CA2 radiatum layer	27
	CA3-Or	CA3 oriens layer	207
	CA3-Rad	CA3 radiatum layer	80
	CEnt	Caudomedial entorhinal cortex	239
	DIEnt	Dorsal intermediate entorhinal cortex	87
	DLEnt	Dorsolateral entorhinal cortex	118
	DS	Dorsal subiculum	37
	GrDG	Granule layer of dentate gyrus	24
	MEnt	Medial entorhinal cortex	21

	MoDG	Molecular layer of dentate gyrus	245
	PaS	Parasubiculum	70
	PoDG	Polymorph layer of dentate gyrus	22
	Post	Postsubiculum	32
	STr	Subiculum transition area	71
	Sub	Subiculum	20
	VIEnt	Ventral intermediate entorhinal cortex	42
**Thalamus**	ADN	Anterior dorsal thalamus nucleus	17
	AM	Anteromedial thalamic nucleus	24
	DLG	Dorsal lateral geniculate nucleus	31
	LD	Laterodorsal thalamic nucleus	37
	LP	Lateral posterior thalamic nucleus	23
	LTN	Lateral thalamus nucleus	20
	MD	Mediodorsal thalamic nucleus	53
	MGV	Medial geniculate nucleus, ventral part	24
	MN	Medial thalamus nucleus	92
	PaXi	Paraxiphoid nucleus of thalamus	30
	PDN	Posterior dorsal thalamus nucleus	70
	PLTN	Posterior lateral thalamic nucleus	39
	Po	Posterior thalamic nuclear group	68
	PV	Paraventricular thalamic nucleus	16
	Re	Reuniens thalamic nucleus	29
	Rt	Reticular nucleus (prethalamus)	78
	VL	Ventrolateral thalamic nucleus	39
	VM	Ventromedial thalamic nucleus	33
	VPL	Ventral posterolateral thalamic nucleus	26
	VPM	Ventral posteromedial thalamic nucleus	53
**Hypothalamus**	Hyp	Hypothalamus	396
	LH	Lateral hypothalamus	59
	MM	Mammillary bodies	19
	Zl	Zona incerta	75
**Midbrain**	APT	Anterior pretectal nucleus	38
	IC	Inferior colliculus	271
	IP	Interpeduncular nucleus	30
	MB	Midbrain	532
	PAG	Periaqueductal gray	208
	PN	Pontine nucleus	34
	SC	Superior colliculus	415
**Hindbrain**	Pons	Pons	796
	SOC	Superior olivary complex	30

Table S2 Behavior-correlated functional connections from common networks.
Each row shows the names of the nodes, the behavioral correlation r and p values (Pearson correlation, two-tailed, uncorrected). $_R$: right hemisphere; $_L$: left hemisphere.

		Node 1	Structure name 1	Node 2	Structure name 2	r	p value
Post day 1	N_{shock}	DLG_L	Dorsal lateral geniculate nucleus	$A24b_L$	Anterior cingulate area, dorsal part	-0.72	0.0018
		$CA3\text{-}Or_L$	CA3 oriens layer	$S1BF_L$	Primary somatosensory cortex, barrel field	-0.68	0.0037
		LS_L	Lateral septum	$CA1\text{-}Rad_L$	CA1 radiatum layer	-0.65	0.0069
		$CA2\text{-}Rad_L$	CA2 radiatum layer	STr_R	Subiculum transition area	-0.58	0.019
		$V1_R$	Primary visual cortex	$S1_R$	Primary somatosensory cortex, unassigned	0.57	0.021
		ADN_L	Anterior dorsal thalamus nucleus	$A24b_L$	Anterior cingulate area, dorsal part	-0.52	0.038
		LP_L	Lateral posterior thalamic nucleus	DEn_L	Dorsal nucleus of the endopiriform	-0.52	0.04
		MN_L	Medial thalamus nucleus	$IPAC_L$	Interstitial nucleus of the posterior limb of the anterior commissure	-0.51	0.041
		$LAcbSh_L$	Accumbens nucleus shell, lateral part	$A24b_L$	Anterior cingulate area, dorsal part	-0.51	0.046
	T_{enter}	$PLTN_L$	Posterior lateral thalamic nucleus	$Pons_R$	Pons	-0.67	0.003
		$V2MM_L$	Secondary visual cortex: mediomedial area	AuD_L	Secondary auditory cortex, dorsal area	-0.67	0.0033
		$V2MM_L$	Secondary visual cortex: mediomedial area	$Au1_L$	Primary auditory cortex	-0.66	0.0038
		$V1_L$	Primary visual cortex	$MPtA_L$	Medial parietal association cortex	-0.65	0.0044
		GP_L	Globus pallidus	$A30_L$	Retrosplenial area, dorsal part	-0.62	0.0077
		PDN_L	Posterior dorsal thalamus nucleus	$Pons_R$	Pons	-0.61	0.0092
		Sub_L	Subiculum	MB_L	Midbrain	0.6	0.01
		$CA1\text{-}Rad_R$	CA1 radiatum layer	Ce_R	Central amygdaloid nucleus	-0.59	0.012
		$V1_R$	Primary visual cortex	$S1_R$	Primary somatosensory cortex, unassigned	-0.58	0.014
		$CA3\text{-}Rad_L$	CA3 radiatum layer	$V1M_R$	Primary visual cortex, monocular area	0.57	0.018
		MGV_R	Medial geniculate nucleus, ventral part	$CEnt_R$	Caudomedial entorhinal cortex	-0.52	0.031
		$CA1\text{-}Rad_L$	CA1 radiatum layer	$MPtA_R$	Medial parietal association cortex	0.52	0.033
		LTN_L	Lateral thalamus nucleus	$S1HL_R$	Primary somatosensory cortex, hindlimb region	0.51	0.034
		ST_L	Bed nucleus of the stria terminalis	$A24b'_L$	Anterior cingulate area, dorsal part	0.51	0.038
		VPM_R	Ventral posteromedial thalamic nucleus	DLG_R	Dorsal lateral geniculate nucleus	-0.51	0.038
		$LAcbSh_L$	Accumbens nucleus shell, lateral part	$A24b_L$	Anterior cingulate area, dorsal part	0.5	0.042
		LS_L	Lateral septum	$VIEnt_L$	Ventral intermediate entorhinal cortex	0.5	0.042
Post day 8	N_{shock}	$CA3\text{-}Or_L$	CA3 oriens layer	$S2_R$	Secondary somatosensory cortex	-0.8	0.0002
		$AcbSh_L$	Accumbens nucleus shell	$A30_L$	Retrosplenial area, dorsal part	-0.6	0.015
		LS_R	Lateral septum	$MEnt_R$	Medial entorhinal cortex	-0.58	0.02
		Rt_L	Reticular nucleus	LD_L	Laterodorsal thalamic nucleus	-0.57	0.02
		$LAcbSh_L$	Accumbens nucleus shell, lateral part	$AcbC_L$	Accumbens nucleus core	-0.52	0.038

	$PLCo_L$	Posterolateral cortical amygdaloid area	$CEnt_R$	Caudomedial entorhinal cortex	-0.52	0.041
T_{enter}	SC_R	Superior colliculus	AM_R	Anteromedial thalamic nucleus	0.6	0.014
	$APir_L$	Amygdalopiriform transition area	Ce_R	Central amygdaloid nucleus	-0.59	0.015
	Zl_R	Zona incerta	DLG_R	Dorsal lateral geniculate nucleus	0.57	0.02
	PAG_R	Periaqueductal gray	LD_R	Laterodorsal thalamic nucleus	0.55	0.028
	LS_R	Lateral septum	$MEnt_R$	Medial entorhinal cortex	0.54	0.032
	PAG_R	Periaqueductal gray	AM_R	Anteromedial thalamic nucleus	0.51	0.046

Table S3 Comparison of top 10 nodes identified by CI and HITS analysis.

This table shows the top 10 ranking nodes according to the mean CI rank under network threshold of $p < 0.05$, $p < 0.01$ and $p < 0.005$ when comparing the 1-Day APA and control (two-sample t-test, two-tailed, uncorrected). R: right hemisphere; L: left hemisphere. The red font is the brain regions identified in both CI and HITS. See Supplementary Table S1 for the abbreviations of brain regions.

**CI**				**HITS**			
**Post day 1**		**Post day 8**		**Post day 1**		**Post day 8**	
**Node**	**Mean Rank**	**Node**	**Mean Rank**	**Node**	**Mean Rank**	**Node**	**Mean Rank**
$Pons_R$	1.0	$MPtA_R$	6.7	$Pons_R$	1.3	LO_R	1.7
$LAcbSh_L$	3.7	PAG_R	7.0	LS_L	5.7	PV_L	2.0
$VIEnt_L$	9.3	LO_R	7.3	$S2_L$	9.0	PV_R	3.3
PN_R	9.7	VM_L	10.7	PaS_R	10.7	$A30_L$	3.7
MB_R	10.0	$S1FL_L$	12.0	CPu_R	15.7	LD_R	6.7
CPu_R	13.0	Hyp_L	14.3	$PMCo_L$	16.0	$A29c_R$	8.0
MM_R	13.7	LD_R	15.3	$MoDG_R$	16.7	$S1Tr_R$	10.3
STr_L	15.3	LS_R	17.0	MGV_L	17.3	PAG_R	13.7
$CEnt_L$	21.7	$LAcbSh_R$	20.0	IP_R	18.3	AM_R	14.7
$S2_L$	22.0	$S1Tr_R$	21.7	$MoDG_L$	23.7	LS_R	15.0

Table S4 The number of animals included in data analysis from each rsfMRI session. Animals that did not show visual responses during the fMRI scan were excluded.

Experimental groups	rsfMRI on post day 1	rsfMRI on post day 8
**1-Day APA**	10 in 12	8 in 12
**1-Day control**	9 in 12	9 in 12
**5-Day APA**	7 in 10	8 in 10
**5-Day control**	5 in 6	5 in 6
