# Supplementary material for: Locating causal hubs of memory consolidation in spontaneous brain network in male mice
Source: Nat Commun. 2023 Sep 5;14:5399. doi: 10.1038/s41467-023-41024-z (PMC10480429; doi:10.1038/s41467-023-41024-z)
Supplement: Supplementary file 3 — Reporting Summary [file 41467_2023_41024_MOESM3_ESM.pdf]

nature portfolio

Corresponding author(s): Kai-Hsiang Chuang

Last updated by author(s): Jul 19, 2023

Reporting Summary

Nature Portfolio wishes to improve the reproducibility of the work that we publish. This form provides structure for consistency and transparency in reporting. For further information on Nature Portfolio policies, see our Editorial Policies and the Editorial Policy Checklist.

Statistics

For all statistical analyses, confirm that the following items are present in the figure legend, table legend, main text, or Methods section.

n/a	Confirmed	
☐	☒	The exact sample size (*n*) for each experimental group/condition, given as a discrete number and unit of measurement
☐	☒	A statement on whether measurements were taken from distinct samples or whether the same sample was measured repeatedly
☐	☒	The statistical test(s) used AND whether they are one- or two-sided<br>*Only common tests should be described solely by name; describe more complex techniques in the Methods section.*
☒	☐	A description of all covariates tested
☐	☒	A description of any assumptions or corrections, such as tests of normality and adjustment for multiple comparisons
☐	☒	A full description of the statistical parameters including central tendency (e.g. means) or other basic estimates (e.g. regression coefficient) AND variation (e.g. standard deviation) or associated estimates of uncertainty (e.g. confidence intervals)
☐	☒	For null hypothesis testing, the test statistic (e.g. F, t, r) with confidence intervals, effect sizes, degrees of freedom and P value noted<br>*Give P values as exact values whenever suitable.*
☒	☐	For Bayesian analysis, information on the choice of priors and Markov chain Monte Carlo settings
☒	☐	For hierarchical and complex designs, identification of the appropriate level for tests and full reporting of outcomes
☐	☒	Estimates of effect sizes (e.g. Cohen's *d*, Pearson's *r*), indicating how they were calculated

Our web collection on statistics for biologists contains articles on many of the points above.

Software and code

Policy information about availability of computer code

Data collection	MRI data were acquired using Paravision 6.0.1 (Bruker GmBH) with custom pulse sequence as described in Lee,et al. NeuroImage 2019. The code for this custom pulse sequence is available from the corresponding author. The AMBMC mouse brain template can be downloaded from https://imaging.org.au/AMBMC/AMBMC.
Data analysis	Data analysis were conducted using MATLAB (Mathworks Inc), Prism (GraphPad Inc) and open source software, including: FSL (https://www.fmrib.ox.ac.uk/fsl), AFNI (https://afni.nimh.nih.gov/), ANTs (http://stnava.github.io/ANTs/), Brain connectivity toolbox (https://sites.google.com/site/bctnet/), Network Based Statistics toolbox (https://sites.google.com/site/bctnet/comparison/nbs), HITS score (https://people.sc.fsu.edu/~jburkardt/m_src/hits/hits.html), Small-worldness (https://github.com/mdhumphries/SmallWorldNess), ComplexCI (https://github.com/zhfkt/ComplexCi/releases), 3D-PCNN (https://sites.google.com/site/chuanglab/software/3d-pcnn), and BrainNet Viewer (https://www.nitrc.org/projects/bnv/).

For manuscripts utilizing custom algorithms or software that are central to the research but not yet described in published literature, software must be made available to editors and reviewers. We strongly encourage code deposition in a community repository (e.g. GitHub). See the Nature Portfolio guidelines for submitting code & software for further information.

Data

Policy information about availability of data

All manuscripts must include a data availability statement. This statement should provide the following information, where applicable:

- Accession codes, unique identifiers, or web links for publicly available datasets
- A description of any restrictions on data availability
- For clinical datasets or third party data, please ensure that the statement adheres to our policy

Supplementary Information/Source Data are provided with this paper. Brain imaging data is available from the corresponding author upon reasonable request. Source data are provided with this paper.

Human research participants

Policy information about studies involving human research participants and Sex and Gender in Research.

Reporting on sex and gender	*n/a*
Population characteristics	*n/a*
Recruitment	*n/a*
Ethics oversight	*n/a*

Note that full information on the approval of the study protocol must also be provided in the manuscript.

Field-specific reporting

Please select the one below that is the best fit for your research. If you are not sure, read the appropriate sections before making your selection.

☒ Life sciences ☐ Behavioural & social sciences ☐ Ecological, evolutionary & environmental sciences

For a reference copy of the document with all sections, see nature.com/documents/nr-reporting-summary-flat.pdf

Life sciences study design

All studies must disclose on these points even when the disclosure is negative.

Sample size	The sample size of 8-10 per group for imaging was based on our previous study in mice (Lee, et al. NeuroImage 2019). Similar sample size was used in other mouse fMRI and behavioral study, such as Shah, et al. Cereb. Cortex 2019.
Data exclusions	*Functional imaging data that did not show visual cortical activation under a visual stimulation indicates unstable neurovascular coupling and hence were excluded from the data analysis. Animals that did not learn the behavioral task during the 5 training trials were also excluded.*
Replication	All experiments were conducted in at least two separate batches of animals to ensure that the findings are not biased by batch to batch difference. No difference between batches were observed.
Randomization	*Animals in a cage were randomly assigned to different behavior, imaging or surgery groups. For animals undergoing experimental procedures in the same day, the order of conducting experiments were also randomized to avoid physiological and behavioral variations due to time of day.*
Blinding	*Data acquisition, postprocessing and analysis were performed blind to the experimental group .*

Behavioural & social sciences study design

All studies must disclose on these points even when the disclosure is negative.

Study description	*Briefly describe the study type including whether data are quantitative, qualitative, or mixed-methods (e.g. qualitative cross-sectional, quantitative experimental, mixed-methods case study).*
Research sample	*State the research sample (e.g. Harvard university undergraduates, villagers in rural India) and provide relevant demographic information (e.g. age, sex) and indicate whether the sample is representative. Provide a rationale for the study sample chosen. For studies involving existing datasets, please describe the dataset and source.*

Sampling strategy	*Describe the sampling procedure (e.g. random, snowball, stratified, convenience). Describe the statistical methods that were used to predetermine sample size OR if no sample-size calculation was performed, describe how sample sizes were chosen and provide a rationale for why these sample sizes are sufficient. For qualitative data, please indicate whether data saturation was considered, and what criteria were used to decide that no further sampling was needed.*
Data collection	*Provide details about the data collection procedure, including the instruments or devices used to record the data (e.g. pen and paper, computer, eye tracker, video or audio equipment) whether anyone was present besides the participant(s) and the researcher, and whether the researcher was blind to experimental condition and/or the study hypothesis during data collection.*
Timing	*Indicate the start and stop dates of data collection. If there is a gap between collection periods, state the dates for each sample cohort.*
Data exclusions	*If no data were excluded from the analyses, state so OR if data were excluded, provide the exact number of exclusions and the rationale behind them, indicating whether exclusion criteria were pre-established.*
Non-participation	*State how many participants dropped out/declined participation and the reason(s) given OR provide response rate OR state that no participants dropped out/declined participation.*
Randomization	*If participants were not allocated into experimental groups, state so OR describe how participants were allocated to groups, and if allocation was not random, describe how covariates were controlled.*

Ecological, evolutionary & environmental sciences study design

All studies must disclose on these points even when the disclosure is negative.

Study description	*Briefly describe the study. For quantitative data include treatment factors and interactions, design structure (e.g. factorial, nested, hierarchical), nature and number of experimental units and replicates.*
Research sample	*Describe the research sample (e.g. a group of tagged Passer domesticus, all Stenocereus thurberi within Organ Pipe Cactus National Monument), and provide a rationale for the sample choice. When relevant, describe the organism taxa, source, sex, age range and any manipulations. State what population the sample is meant to represent when applicable. For studies involving existing datasets, describe the data and its source.*
Sampling strategy	*Note the sampling procedure. Describe the statistical methods that were used to predetermine sample size OR if no sample-size calculation was performed, describe how sample sizes were chosen and provide a rationale for why these sample sizes are sufficient.*
Data collection	*Describe the data collection procedure, including who recorded the data and how.*
Timing and spatial scale	*Indicate the start and stop dates of data collection, noting the frequency and periodicity of sampling and providing a rationale for these choices. If there is a gap between collection periods, state the dates for each sample cohort. Specify the spatial scale from which the data are taken*
Data exclusions	*If no data were excluded from the analyses, state so OR if data were excluded, describe the exclusions and the rationale behind them, indicating whether exclusion criteria were pre-established.*
Reproducibility	*Describe the measures taken to verify the reproducibility of experimental findings. For each experiment, note whether any attempts to repeat the experiment failed OR state that all attempts to repeat the experiment were successful.*
Randomization	*Describe how samples/organisms/participants were allocated into groups. If allocation was not random, describe how covariates were controlled. If this is not relevant to your study, explain why.*
Blinding	*Describe the extent of blinding used during data acquisition and analysis. If blinding was not possible, describe why OR explain why blinding was not relevant to your study.*

Did the study involve field work? ☐ Yes ☐ No

Field work, collection and transport

Field conditions	*Describe the study conditions for field work, providing relevant parameters (e.g. temperature, rainfall).*
Location	*State the location of the sampling or experiment, providing relevant parameters (e.g. latitude and longitude, elevation, water depth).*
Access & import/export	*Describe the efforts you have made to access habitats and to collect and import/export your samples in a responsible manner and in compliance with local, national and international laws, noting any permits that were obtained (give the name of the issuing authority, the date of issue, and any identifying information).*

Disturbance | *Describe any disturbance caused by the study and how it was minimized.*

Reporting for specific materials, systems and methods

We require information from authors about some types of materials, experimental systems and methods used in many studies. Here, indicate whether each material, system or method listed is relevant to your study. If you are not sure if a list item applies to your research, read the appropriate section before selecting a response.

Materials & experimental systems

n/a	Involved in the study
☒	☐ Antibodies
☒	☐ Eukaryotic cell lines
☒	☐ Palaeontology and archaeology
☐	☒ Animals and other organisms
☒	☐ Clinical data
☒	☐ Dual use research of concern

Methods

n/a	Involved in the study
☒	☐ ChIP-seq
☒	☐ Flow cytometry
☐	☒ MRI-based neuroimaging

Antibodies

Antibodies used | *Describe all antibodies used in the study; as applicable, provide supplier name, catalog number, clone name, and lot number.*

Validation | *Describe the validation of each primary antibody for the species and application, noting any validation statements on the manufacturer's website, relevant citations, antibody profiles in online databases, or data provided in the manuscript.*

Eukaryotic cell lines

Policy information about cell lines and Sex and Gender in Research

Cell line source(s) | *State the source of each cell line used and the sex of all primary cell lines and cells derived from human participants or vertebrate models.*

Authentication | *Describe the authentication procedures for each cell line used OR declare that none of the cell lines used were authenticated.*

Mycoplasma contamination | *Confirm that all cell lines tested negative for mycoplasma contamination OR describe the results of the testing for mycoplasma contamination OR declare that the cell lines were not tested for mycoplasma contamination.*

Commonly misidentified lines (See ICLAC register) | *Name any commonly misidentified cell lines used in the study and provide a rationale for their use.*

Palaeontology and Archaeology

Specimen provenance | *Provide provenance information for specimens and describe permits that were obtained for the work (including the name of the issuing authority, the date of issue, and any identifying information). Permits should encompass collection and, where applicable, export.*

Specimen deposition | *Indicate where the specimens have been deposited to permit free access by other researchers.*

Dating methods | *If new dates are provided, describe how they were obtained (e.g. collection, storage, sample pretreatment and measurement), where they were obtained (i.e. lab name), the calibration program and the protocol for quality assurance OR state that no new dates are provided.*

☐ Tick this box to confirm that the raw and calibrated dates are available in the paper or in Supplementary Information.

Ethics oversight | *Identify the organization(s) that approved or provided guidance on the study protocol, OR state that no ethical approval or guidance was required and explain why not.*

Note that full information on the approval of the study protocol must also be provided in the manuscript.

Animals and other research organisms

Policy information about studies involving animals; ARRIVE guidelines recommended for reporting animal research, and Sex and Gender in Research

Laboratory animals	Adult C57BL/6 mice were purchased from the Animal Resources Centre, WA, Australia. The animal was kept on a 12 h–12 h light-dark cycle at 20-22 degree C and 40-60% humidity. Animals were housed up to five per cage and were provided ad libitum access to water and food. Experiments were conducted at 10-16 weeks old.
Wild animals	*No wild animal was used.*
Reporting on sex	*Only male mice was used in this study to avoid the effects of estrus cycle on behavior, particularly spatial learning and memory, and functional connectivity of the brain. It is well established that estrogen affects the hippocampal morphology, plasticity, spatial memory and stress involved in aversive learning (Frick, et al. Learn Mem 2015; Pompili, et al. Behav Brain Res 2010; Hokenson, et al. J Neurosci 2021). Furthermore, menstrual cycle is found to affect functional connectivity measured in the spontaneous brain activity in humans (Arelin, et al. Front Neurosci 2015; Hidalgo-Lopez, et al. Brain Struct Funct 2020). So far, mouse fMRI has been predominantly conducted in male mice. Thus estrogen effect on mouse brain network remains to be elucidated. As the study focuses on the relationship between spontaneous network activity and memory formation, estrogen or estrus cycle could be a strong covariate leading to sex difference in the results. Further research would be needed to understand the sex effects on brain network plasticity.*
Field-collected samples	*No field-collected smaple was used.*
Ethics oversight	All experimental procedures were approved by the Animal Ethics Committee of the University of Queensland and conducted in compliance with the Queensland Animal Care and Protection Act 2001 and the Australian Code of Practice for the Care and Use of Animals for Scientific Purposes.

Note that full information on the approval of the study protocol must also be provided in the manuscript.

Clinical data

Policy information about clinical studies
All manuscripts should comply with the ICMJE guidelines for publication of clinical research and a completed CONSORT checklist must be included with all submissions.

Clinical trial registration	*Provide the trial registration number from ClinicalTrials.gov or an equivalent agency.*
Study protocol	*Note where the full trial protocol can be accessed OR if not available, explain why.*
Data collection	*Describe the settings and locales of data collection, noting the time periods of recruitment and data collection.*
Outcomes	*Describe how you pre-defined primary and secondary outcome measures and how you assessed these measures.*

Dual use research of concern

Policy information about dual use research of concern

Hazards

Could the accidental, deliberate or reckless misuse of agents or technologies generated in the work, or the application of information presented in the manuscript, pose a threat to:

No	Yes	
☐	☐	Public health
☐	☐	National security
☐	☐	Crops and/or livestock
☐	☐	Ecosystems
☐	☐	Any other significant area

Experiments of concern

Does the work involve any of these experiments of concern:

No	Yes	
☐	☐	Demonstrate how to render a vaccine ineffective
☐	☐	Confer resistance to therapeutically useful antibiotics or antiviral agents
☐	☐	Enhance the virulence of a pathogen or render a nonpathogen virulent
☐	☐	Increase transmissibility of a pathogen
☐	☐	Alter the host range of a pathogen
☐	☐	Enable evasion of diagnostic/detection modalities
☐	☐	Enable the weaponization of a biological agent or toxin
☐	☐	Any other potentially harmful combination of experiments and agents

ChIP-seq

Data deposition

☐ Confirm that both raw and final processed data have been deposited in a public database such as GEO.

☐ Confirm that you have deposited or provided access to graph files (e.g. BED files) for the called peaks.

Data access links<br>*May remain private before publication.*	*For "Initial submission" or "Revised version" documents, provide reviewer access links. For your "Final submission" document, provide a link to the deposited data.*
Files in database submission	*Provide a list of all files available in the database submission.*
Genome browser session<br>(e.g. UCSC)	*Provide a link to an anonymized genome browser session for "Initial submission" and "Revised version" documents only, to enable peer review. Write "no longer applicable" for "Final submission" documents.*

Methodology

Replicates	*Describe the experimental replicates, specifying number, type and replicate agreement.*
Sequencing depth	*Describe the sequencing depth for each experiment, providing the total number of reads, uniquely mapped reads, length of reads and whether they were paired- or single-end.*
Antibodies	*Describe the antibodies used for the ChIP-seq experiments; as applicable, provide supplier name, catalog number, clone name, and lot number.*
Peak calling parameters	*Specify the command line program and parameters used for read mapping and peak calling, including the ChIP, control and index files used.*
Data quality	*Describe the methods used to ensure data quality in full detail, including how many peaks are at FDR 5% and above 5-fold enrichment.*
Software	*Describe the software used to collect and analyze the ChIP-seq data. For custom code that has been deposited into a community repository, provide accession details.*

Flow Cytometry

Plots

Confirm that:

☐ The axis labels state the marker and fluorochrome used (e.g. CD4-FITC).

☐ The axis scales are clearly visible. Include numbers along axes only for bottom left plot of group (a 'group' is an analysis of identical markers).

☐ All plots are contour plots with outliers or pseudocolor plots.

☐ A numerical value for number of cells or percentage (with statistics) is provided.

Methodology

Sample preparation	*Describe the sample preparation, detailing the biological source of the cells and any tissue processing steps used.*
Instrument	*Identify the instrument used for data collection, specifying make and model number.*

Software | *Describe the software used to collect and analyze the flow cytometry data. For custom code that has been deposited into a community repository, provide accession details.*

Cell population abundance | *Describe the abundance of the relevant cell populations within post-sort fractions, providing details on the purity of the samples and how it was determined.*

Gating strategy | *Describe the gating strategy used for all relevant experiments, specifying the preliminary FSC/SSC gates of the starting cell population, indicating where boundaries between "positive" and "negative" staining cell populations are defined.*

☐ Tick this box to confirm that a figure exemplifying the gating strategy is provided in the Supplementary Information.

Magnetic resonance imaging

Experimental design

Design type | *Resting-state fMRI*

Design specifications | *BOLD fMRI was acquired using a single-shot multiband EPI sequence.*

Behavioral performance measures | *None.*

Acquisition

Imaging type(s) | *BOLD fMRI*

Field strength | *9.4T*

Sequence & imaging parameters | *Single-shot multiband gradient-echo EPI with TR=300ms, TE=15ms, flip angle=30 degree, 4 bands, inplane-resolution=0.3x0.3mm^2, 16 slices, slice thickness=0.5mm, gap=0.1mm. Repetition=2000.*

Area of acquisition | *Entire cerebrum and part of the cerebellum.*

Diffusion MRI ☐ Used ☒ Not used

Preprocessing

Preprocessing software | *FSL mcflirt was used for motion correction,*

Normalization | *Nonlinear registration to the AMBMC template using ANTs.*

Normalization template | *Customed built EPI brain template coregistered to the AMBMC MRI brain template space (https://imaging.org.au/AMBMC/AMBMC).*

Noise and artifact removal | *Nuisance removal was conducted using motion parameters and 10 principal components of signals outside the brain, based on Chuang, et al. NeuroImage 2019. Temporal filter at 0.01-0.3 Hz and spatial Gaussian smoothing of 0.6mm were also applied.*

Volume censoring | *None.*

Statistical modeling & inference

Model type and settings | *none.*

Effect(s) tested | *none.*

Specify type of analysis: ☐ Whole brain ☐ ROI-based ☒ Both

Statistic type for inference (See Eklund et al. 2016) | *Seed-based functional connectivity, measured by Pearson's correlation, among 230 ROIs across the whole brain. Two-sample t-test was used to determine behavioral-dependent connectivity changes. Independent component analysis (30 components) with dual regression.*

Correction | *FDR correction, cluster-wise correction, and permutation tests.*

Models & analysis

n/a	Involved in the study	
☐	☒	Functional and/or effective connectivity
☐	☒	Graph analysis
☒	☐	Multivariate modeling or predictive analysis

Functional and/or effective connectivity

Pearson's correlation. Regression (ICA-dual regression)

Graph analysis

Global efficiency, modularity, transitivity, centrality, HITS score, and giant component size were calculated from weighted and binarized connectivity matrix, compared over a range of t-value thresholds. Small-world network properties were compared over a range of z thresholds. The area-under-the-curve was used to compare with the null distribution of those from random networks to determine the statistical significance.

Multivariate modeling and predictive analysis

none.
